# Shaping epithelial lumina under pressure

**DOI:** 10.1042/BST20230632C

**Published:** 2024-02-28

**Authors:** Matthew J. Bovyn, Pierre A. Haas

**Affiliations:** 1 Max Planck Institute for the Physics of Complex Systems, Nöthnitzer Straße 38, 01187 Dresden, Germany; 2 Max Planck Institute of Molecular Cell Biology and Genetics, Pfotenhauerstraße 108, 01307 Dresden, Germany; 3 Center for Systems Biology Dresden, Pfotenhauerstraße 108, 01307 Dresden, Germany

**Keywords:** biophysics, cell mechanics, epithelial cells, lumen formation

## Abstract

The formation of fluid- or gas-filled lumina surrounded by epithelial cells pervades development and disease. We review the balance between lumen pressure and mechanical forces from the surrounding cells that governs lumen formation. We illustrate the mechanical side of this balance in several examples of increasing complexity, and discuss how recent work is beginning to elucidate how nonlinear and active mechanics and anisotropic biomechanical structures must conspire to overcome the isotropy of pressure to form complex, non-spherical lumina.

## Introduction

Lumina are liquid- or gas-filled spaces surrounded and sealed off by cells. They are ubiquitous in the body plans of metazoans. In large organisms, tube-shaped lumina allow material to flow through, enabling transport and exchange of material constituents such as oxygen from air and nutrients from food. The diversity of lumen structures is mirrored by the diversity in the ways in which they are formed [1–3] and the diversity of the mechanical forces [4] and molecular mechanisms [5] involved in their formation. Some of the lumina that are formed during development are persistent, eventually growing into tube-shaped structures such as the gut or blood vessels, while others are transient, such as the mammalian blastocoel. Because lumina are sealed, they are able to support fluid pressure, and indeed, many lumina have been found to be pressurised [6], including the mouse blastocoel [7, 8], the chick brain [9], the zebrafish otic vesicle [10], and the developing lung [11–13]. In addition to these *in vivo* instantiations of pressurised lumina, cultured cells in various conditions also form pressurised lumina. Examples include Madin–Darby canine kidney (MDCK) cells in three-dimensional (3D) culture [14–16] and in engineered two-dimensional (2D) culture [17], and mouse hepatocytes [18–21]. Proper pressurisation is important, as it is required for correct organ development in some systems [13] and is more generally thought to drive lumen opening [20].

Here, we review recent progress on the biophysics of lumen formation and lumen shaping that complements the more established understanding of the cell biology of these systems. We will focus on how simple physical models can inform our understanding of the formation of pressurised spherical lumina and of their symmetry breaking to define more complex lumen shapes.

We begin by introducing the cell biological concepts that underpin lumen formation and these physical models via two examples, MDCK cells grown in 3D culture and hepatocytes forming bile canaliculi. In both systems, lumina are formed by pumping of liquid into a sealed extracellular space in a process sometimes called cord hollowing [1, 2, 22, 23]. Because these systems are tractable *in vitro*, because the complex cell biology of apico-basal polarity establishment in epithelia is inexorably tied to lumen formation [22, 24], and because of the physiological importance of the kidney and liver lumenal systems, these epithelial models have been studied intensely.

We reiterate that epithelia can also form lumina by various other mechanisms [1–3] and add that endothelial cells also form lumina by somewhat similar mechanisms [23]. While we shall not review these in detail, the mechanical principles discussed here may apply to these systems, too.

### Example system 1: MCDK cysts

**Figure 1.**
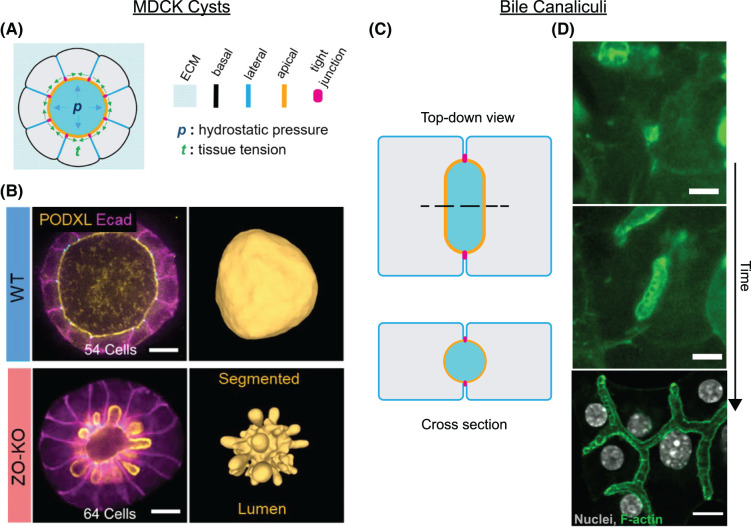
Examples of pressurised epithelial lumina. (**A**) MDCK cysts have a typical epithelial cell polarisation, with apical surfaces facing the lumen and basal surfaces opposite, facing the surrounding matrix, and lateral surfaces at cell–cell interfaces. Tight junctions separate the apical and lateral surfaces. (**B**) The lumina of MDCK cysts can take on a variety of shapes depending on culture conditions and genetic perturbations: Examples of a wild-type cyst and a Zonula occludens-1 and Zonula occludens-2 double knockout (ZO-KO) [16]. The wild-type cyst is spherical, while in the ZO-KO the apical cell surfaces bulge out toward the basal side. Scale bar: 10 μm. (**C**) Bile canaliculi lumina are formed by just two hepatocytes. The apical cortices of the two cells surround most of the lumen, with the junctional complex located at the cell–cell interface. (**D**) Growth of bile canaliculi: Nascent bile canaliculi are spherical lumina that elongate to form the bile canaliculi network [29]. Striations across the tubular bile canalculi are ‘apical bulkheads’. Scale bar: 10 μm. (**A**) and (**B**) have been modified from [16] under a CC-BY-NC license, and (**D**) has been modified from [29] under a CC-BY license.

MDCK cell cultures have long been used to study the process of apico-basal polarisation in which epithelial cells determine their apical and baso-lateral surfaces [25–27]. Many-celled cysts surrounding a fluid lumen can form in MDCK cell culture embedded in matrix [1, 16, 28]. In these cysts, the apical cell surfaces face the lumen, the lateral sides face the other cells, and the basal surfaces face the matrix (Figure 1A). The apical surface is separated from the basal and lateral surfaces by junction complexes which serve both to seal the lumen and to partition the apical domain of the plasma membrane [2, 27]. The junction complexes consist of tight junctions, which act to seal the lumen against the flow of molecules, and adherens junctions, which provide the force to hold cells together [2, 27, 30]. The apical cortex is also rich in actomyosin, in particular near the junctions, serving as a mechanical contractile element in the overall tissue structure [30, 31]. These structural elements of MDCK cysts underline that apical surfaces, junctions, lateral cell–cell interfaces, and basal surfaces all have different mechanical properties that, together, determine the mechanics of the lumen.

Recent studies have revealed that MDCK cysts can adopt a rich variety of shapes: While the lumen is usually spherical to a good approximation (Figure 1B), the apical cell surfaces may also strongly protrude into the lumen [15] or into the cell interior [16], depending on culture conditions and genetic perturbations (Figure 1B). These studies thus highlight the complex interplay between anisotropic cell mechanics and lumen pressure, and illustrate how lumina can form at a range of pressures, both below and above cytoplasmic pressure. Furthermore, mechanical models in both studies [15, 16] have suggested a surprising role for apical area conservation in setting the overall lumen shape, a phenomenon with neither a clear underlying cell biological mechanism nor an obvious mechanical origin. Elucidating the basis for these noncanonical lumen shapes is, therefore, a problem of both cell biological and mechanical interest.

### Example system 2: bile canaliculus growth

Hepatocytes from mice and rats also form pressurised lumina in culture [21, 32, 33]. *In vivo*, these structures extend and connect to form the bile canaliculi network [34, 35], which transports bile from the hepatocytes that produce it to the bile ducts which eventually drain the bile into the intestine. As in MDCK cysts, the apical surfaces of hepatocytes face the lumen [36]. However, hepatocyte lumina differ from those of MDCK cells in many ways [37]. Foremost, hepatocyte lumina are bicellular: They are formed by the apical surfaces of just two cells (Figure 1C). The mechanics of the lumen are, therefore, dominated by the mechanics of the apical surfaces of these two cells. When first formed (Figure 1D), bile canaliculi lumina are roughly spherical [29, 35], in contrast with their eventual tubular structure. The transition from spherical to tubular shapes occurs during their early development (Figure 1D). The subsequent direction of elongation is known to be guided by external cues and elongation itself can be explained by the balance of mechanical forces determined by the geometry of the junctions [18]. A more detailed analysis of the physics of this system has yielded general insights into lumen dynamics [20].

Structures called ‘apical bulkheads’ can be found in bile canaliculi after the transition into a tube [29]. While these bulkheads are known to be mechanical elements that increase the pressure that a bile canaliculus can hold [21], the biological and physical mechanisms of their formation remain a mystery. Understanding more about how bile canaliculi transition to a tubular shape and how apical bulkheads form, therefore, promises key insights into the formation of bile canaliculi in development and into the response of bile canaliculi to bile pressure changes in normal physiology and in disease [38].

Because of the biological differences between even just these two example systems, one cannot expect to predict their mechanics as simply as one would predict those of a soap bubble or a rubber balloon. Nonetheless, to understand the mechanical principles underpinning lumen formation, it is still instructive to outline the mechanics of these simpler ‘lumina’. In the following sections, we, therefore, move from these basic mechanical models to slightly more complex ones that capture more of the humongous mechanical complexity of cells, their cortices, and of cell–cell interfaces. In this way, we will review the current understanding of the mechanics of pressurised lumina. We will emphasise in particular that even such simple models can recapitulate the qualitative behaviour of more complex models of the lumen and cell mechanics, which demonstrates that these simple models can still be useful to highlight the essential physics even in light of the complex reality of cell mechanics.

## Mechanics of spherical lumina

We first discuss the ‘spherical cow’ of lumen formation, i.e., the basic problem of a spherical, pressurised lumen surrounded by cellular material. As discussed in detail in [39], the lumen grows or shrinks due to fluid entering or leaving it due to mechanical, electrochemical, and active effects combining to yield a total flux

1 $$J=J_{\text{mechanical}}+J_{\text{electrochemical}}+J_{\text{active}},\;\left(1\right)$$

in which $J_{\text{mechanical}}=-\lambda p$, where p is the mechanical pressure difference between the inside and outside of the lumen and the proportionality constant λ is the permeability of the surrounding cellular material to water. The pressure difference p is set, as discussed in detail below, by the mechanics of the surrounding cellular material. The electrochemical flux $J_{\text{electrochemical}}$ includes a contribution from the osmotic pressure difference resulting from solute concentration imbalances between the inside and outside of the lumen, and, in general, the additional contributions from solute flows allowed by linear irreversible thermodynamics [39]. Finally, $J_{\text{active}}$ summarises additional transport due to cellular processes. While we do not believe that any direct active water transporter is known at present, transport, and docking of vesicles to the apical surfaces could contribute to this flux. The physics of the lumen dynamics that arise for imbalanced fluxes, J≠0, have been explored, for example, in [20, 40].

Here, we focus on the mechanical forces in lumen formation and hence $J_{\text{mechanical}}$, and refer to the comprehensive and excellent review on tissue hydraulics by Torres-Sánchez et al. [39] for further details about the electrochemical contribution $J_{\text{electrochemical}}$ to J.

The most basic mechanical question is: How do the mechanics of the cells surrounding the lumen set the pressure difference p? Mechanical equilibrium requires the pressure p to be balanced by forces generated by the cells surrounding the lumen. In lumina surrounded by many cells, exemplified by the MDCK cysts discussed above, the mechanical properties of the cells (both internal and at their surfaces), their contractility, and the properties of the cell–cell interfaces combine to determine the effective mechanics at the scale of the lumen and hence the forces which resist lumen expansion and, by extension, p. In bicellular lumina, such as nascent bile canaliculi, the mechanics and contractility of the apical cortices, cell–cell interfaces, and cell interiors similarly determine the mechanical forces against which pressure acts. In the following, we refer to the mechanical structure surrounding the lumen in either of these cases as the *mechanical surface*.

### The Young–Laplace law

**Figure 2.**
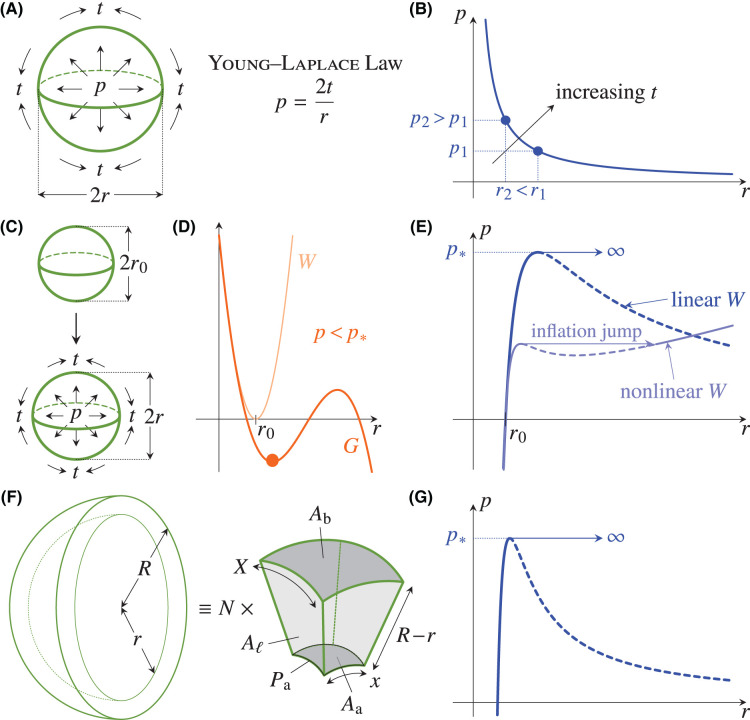
Mechanics of spherical lumina. (**A**) The Young–Laplace law relates the pressure p inflating a spherical surface of radius r to the tension t in the surface. (**B**) Plot of the pressure–radius relationship predicted by the Young–Laplace law at constant tension t: If a lumen of pressure $p_{1}$ has radius $r_{1}$, then a lumen of larger pressure $p_{2}>p_{1}$ has smaller radius $r_{2}<r_{1}$. (**C**) Elastic model of lumen inflation: A spherical lumen of undeformed radius $r_{0}$ inflates to one of radius r under an imposed pressure p. (**D**) The linear elastic energy W of the system has a minimum at $r=r_{0}$, while the radius of the lumen is set by the minimum (highlighted point) of the enthalpy G, which is given in eqn (3). This minimum exists for $p<p_{*}$. (**E**) The resulting pressure–radius relation is given by eqn (4), and has a maximum at $p_{*}$; for $p>p_{*}$, the lumen radius diverges, r→∞. This divergence is replaced by an inflation jump of finite size if the linear elastic energy density is replaced with a nonlinear Mooney–Rivlin density [42, 43]. (**F**) Mean-field model of a spherical cyst of inner radius r and outer radius R, consisting of N identical cells which are incompressible square frusta of inner side x, outer side X, and height R−r. Their apical area $A_{\text{a}}$, basal area $A_{\text{b}}$, lateral area $A_{\ell }$, and apical perimeter $P_{\text{a}}$ define the mechanical energy W of the cyst. (**G**) The pressure–radius relation that results from this model in the limit of a large cyst of many cells, expressed by eqn (7), is qualitatively similar to that of the elastic model, but differs at a quantitative level, because of the emergent nature of the mechanics at the cyst scale.

The balance between the lumen pressure and the tension in the mechanical surface required by mechanical equilibrium is expressed by the Young–Laplace law [41] that links pressure, tension, and curvature: In the simplest case of a spherical lumen of radius r (Figure 2A), let t denote the tension in the mechanical surface of the lumen. Expanding the lumen to a radius r+δr, i.e., expanding its surface from $4\pi r^{2}$ to $4\pi (r+\delta r{)}^{2}$ requires an energy $t[4\pi (r+\delta r{)}^{2}-4\pi r^{2}]\approx 8\pi tr\;\delta r$. This is provided by the work done by the pressure in expanding the lumen to this radius, i.e., from a volume $\frac{4}{3}\pi \;r^{3}$ to a volume $\frac{4}{3}\pi \;(r+\delta r{)}^{3}$, which is $p[\frac{4}{3}\pi \;(r+\delta r{)}^{3}-\frac{4}{3}\pi \;r^{3}]\approx 4\pi pr^{2}\delta r$. It follows that

2 $$p=\frac{2t}{r}.\;\left(2\right)$$

More generally, the Young–Laplace law states that p=2tH, where H is the mean curvature of the surface [41]. The tension t is set by the mechanical properties and the deformation of the mechanical surface. In what follows, we illustrate this mechanical balance and the effect of these mechanical properties by simple example calculations.

### Example 1: soap bubble

The simplest case has a constant tension t, independent of the radius r of the lumen, in which case the physics become those of a soap bubble: eqn (2) shows that the larger the radius r of lumen, the smaller the pressure difference p (Figure 2B). The assumption of constant t, therefore, contradicts the expectation that the lumen inflate with increasing pressure, in the same way as a rubber balloon would inflate on oral pressurisation.

### Constitutive relations

This contradiction can be resolved by describing the mechanical surface as one would describe a rubber balloon, i.e., using an elastic model. This imposes a relation between the tension t and the geometry and deformation of the lumen that, as illustrated below, follows the intuition that increasing pressure should increase the size of the lumen.

More generally, the relation between t and lumen geometry and deformation are expressed by the *constitutive relations* describing the material that constitutes the mechanical surface. On short timescales, the cell cortices and other mechanical elements constituting the mechanical surface are expected to deform proportionally to small external forces, defining an elastic response [31]. On longer timescales, the mechanical surface is expected to turn over. For example, the cortex which dominates the mechanics of the bile canaliculi turns over due to (de)polymerisation of actin filaments in the cytoskeleton on a timescale of ∼10 s [31]. This suggests a more fluid behaviour of the mechanical surface beyond this turnover timescale, calling for a transiently viscous, or more generally, a viscoelastic description [44]. In systems in which the mechanical surface is determined by many cells, the timescale of junction turnover, of the order of minutes [45], and the timescale of stress dissipation by cell rearrangements, of the order of hours [46], similarly contribute to the turnover timescale of the mechanical surface.

### Example 2: elastic balloon

We now illustrate in more detail how an elastic description resolves the previous contradiction. Such a description involves the radius $r_{0}$ and thickness $h\ll r_{0}$ of the undeformed mechanical surface, and the radius r that it deforms to under the imposed pressure (Figure 2C). This mechanical problem of the inflation of an elastic balloon has a long history [42, 43, 47], so the following calculation is likely folklore: The deformation of the balloon is described by the strains $\varepsilon_{∥}=r/r_{0}-1$ in the plane of the mechanical surface, and $\varepsilon_{⊥}$ in the direction perpendicular to it. Assuming the material to be incompressible for the sake of simplicity, it follows that $\varepsilon_{⊥}=-2\varepsilon_{∥}$. The simplest choice for the energy density of the mechanical surface is the linear elastic density $w=C(2\varepsilon_{∥}^{2}+\varepsilon_{⊥}^{2})$, where C is a material parameter, and so its elastic energy is $W=4\pi r_{0}^{2}hw=24\pi Ch(r-r_{0}{)}^{2}$ (Figure 2D). At fixed pressure p, the mechanical surface minimises its enthalpy $G=W-p(\frac{4}{3}\pi r^{3})$ (Figure 2D), wherein the factor in parentheses is the lumen volume. Thus

3 $$G=24\pi Ch(r-r_{0}{)}^{2}-\frac{4\pi }{3}pr^{3}.\;\left(3\right)$$

If $p<p_{*}\equiv 3Ch/r_{0}$, G has a local minimum at a radius r given implicitly by

4 $$p=\frac{12Ch}{r}\left(1-\frac{r_{0}}{r}\right).\;\left(4\right)$$

By comparison with eqn (2), this shows that $t=6Ch(1-r_{0}/r)$, which is now not a constant. At small pressures, it increases with increasing r to allow the lumen radius to increase with increasing pressure (Figure 2E). As pressures and thus deformations increase, the assumptions of a linear elastic description are stretched thin. If we nonetheless continue on to $p>p_{*}$, there is no such local minimum and r→∞ minimises G. This divergence corresponds to expansion into an infinitely large and thin lumen. We note that this does not correspond to rupture, the physics of which are not included in this description. This behaviour is rather a consequence of the linear energy density w invoked here. It can be regularised by replacing w with a nonlinear energy density that can describe larger deformations and hence the response to larger p. One example of such a density is a so-called Mooney–Rivlin density [43] which can feature an inflation jump discontinuity [42, 43] that is a remnant of this divergence (Figure 2E).

This Mooney–Rivlin density is only one possible choice of nonlinear elastic energy density. In general, the nonlinear rheological properties of cells and tissues are neither universal nor well understood [44, 48–51]. This is because the laws governing the mechanical surface of the lumen are emergent: They arise from the mechanics of the cells surrounding the lumen, including those of their cortices and junctions. They are, therefore, *a priori* different from the elastic mechanics presented above.

### Example 3: mean-field vertex model

Inspired by the intriguing anisotropic structural properties of cells in MDCK cysts and related epithelial tissues, a large body of work has, therefore, developed discrete vertex models of epithelial cell sheets [52–55] to capture their specific mechanical behaviour. These have been applied, in particular, to the description of lumen mechanics [56–59]. In these models, cells are represented as polyhedra, the faces and edges of which are endowed with different tensions that represent, for example, cortex mechanics. One incarnation of these vertex models thus associates with each cell an energy

5 $$w=\Gamma_{a}A_{a}+\Gamma_{b}A_{b}+\Gamma_{\ell }A_{\ell }+\Lambda P_{a},\;\left(5\right)$$

where $A_{a},A_{b},A_{\ell }$ are the apical, basal, and lateral surface areas of the cell, respectively, $P_{a}$ is its apical perimeter, $\Gamma_{a},\Gamma_{b}>0,\Gamma_{\ell }≷0$ are the anisotropic tensions of the apical, basal, and lateral sides, and Λ is the apical belt tension. Thus $\Gamma_{a}\neq \Gamma_{b}$ imposes apico-basal polarity. If $\Gamma_{\ell }>0$, tension in the lateral sides dominates over cell–cell adhesion, while the latter dominates if $\Gamma_{\ell }<0$ [53]. The cells are incompressible, and have volume V.

Here, we will develop a mean-field description of this vertex model to provide a simple model of lumen mechanics and to highlight similarities and surprising differences to the elastic description developed above. We thus consider a spherical lumen of radius r surrounded by N cells to form a cyst of outer radius R (Figure 2F). In our mean-field description, each of these cells is an incompressible square frustum of volume V, inner side x, outer side X, and height R−r, subtending an angle α=x/r=X/R (Figure 2F). The biologically somewhat implausible choice of square frusta is one of mathematical convenience, as our emphasis here is on simple calculations that illustrate mechanical principles rather than accurate descriptions of specific cellular systems. Each cell, therefore, has apical perimeter $P_{a}=4x$ and lateral surface area $A_{\ell }=4\{\alpha /(2\pi )[\pi (R^{2}-r^{2})]\}=2\alpha (R^{2}-r^{2})$. Calculation of the apical and basal areas of these cells is an exercise in spherical geometry that leads to $A_{a}/r^{2}=A_{b}/R^{2}=4\cos^{-1}\left(\cos^{1/2}\alpha \sec^{2}\left(\alpha /2\right)\right)$. The geometric quantities x,X,R are determined in terms of r by the requirement that N cells cover the lumen and cyst surfaces, and fill the volume in between them, viz. $4\pi r^{2}=NA_{a}$, $4\pi R^{2}=NA_{b}$, $\frac{4}{3}\pi (R^{3}-r^{3})=NV$. The mechanical energy of the cyst is then, upon some trigonometric gymnastics, found to be

6 $$\begin{array}{rl} W=Nw & =4\pi \Gamma_{a}r^{2}+4\pi \Gamma_{b}{\left(r^{3}+\frac{3NV}{4\pi }\right)}^{2/3}\\ & \;+N\left\{2\Gamma_{\ell }\left[{\left(r^{3}+\frac{3NV}{4\pi }\right)}^{2/3}-r^{2}\right]+4\Lambda r\right\}\cos^{-1}{\left(\sec\frac{\pi }{N}-\tan\frac{\pi }{N}\right)}^{2}. \end{array}\;\left(6\right)$$

In the limit of a large cyst of many cells, $r\gg (3NV/4\pi {)}^{1/3}$ and N≫1, and this approximates to

7 $$W\approx 4\pi \left[(\Gamma_{a}+\Gamma_{b})r^{2}+\frac{\Gamma_{\ell }}{2}{\left(\frac{N}{\pi }\right)}^{3/2}\frac{V}{r}+2\Lambda {\left(\frac{N}{\pi }\right)}^{1/2}r\right].\;\left(7\right)$$

Minimising the enthalpy $G=W-\frac{4}{3}\pi pr^{3}$ in the tension-dominated case $\Gamma_{\ell }>0$ then yields, again, an implicit equation for the lumen radius r,

8 $$p=\frac{2}{r}\left[(\Gamma_{a}+\Gamma_{b})+{\left(\frac{N}{\pi }\right)}^{1/2}\frac{\Lambda }{r}-{\left(\frac{N}{\pi }\right)}^{3/2}\frac{\Gamma_{\ell }V}{4r^{3}}\right],\;\left(8\right)$$

in which, by comparison with eqn (2), the term in the square brackets is the tension of the mechanical surface: The microscopic properties of the cells and the geometry of the cyst conspire to set this emergent tension. This has the same qualitative behaviour as the elastic result (4): Again, there exists a critical pressure $p_{*}$ above which this equation has no solution (Figure 2G), because the vertex model (5) lacks the nonlinearities that prevent the cells from thinning indefinitely. One example of such a nonlinearity might be the finite compressibility of the cell nuclei that has been included in the model of Hannezo *et al.* [53].

Importantly, this model and the elastic model differ at a quantitative level: There is no mapping of eqn (8) onto the elastic result (4). This emphasises that the mechanics that emerge from such a model are in general different from linear elasticity. Indeed, the continuum limit of even a simple discrete vertex model can include non-elastic terms [60].

## Mechanics of non-spherical lumina

The ‘spherical-cow approximation’ beloved of physicists that has pervaded the previous section and indeed much previous work on lumenal mechanics cannot of course describe the complex, non-spherical lumen shapes in biological systems. In particular, many mature lumina are tubes. Understanding the emergence of such shapes requires understanding how a growing, spherical lumen breaks its symmetry to form an elongating, tubular lumen.

Pressure is the same in all directions. This isotropy implies that the emergence of non-spherical shapes requires mechanical anisotropy at the scale of the lumen or spontaneous symmetry breaking of a mechanically isotropic lumen.

This symmetry breaking could indeed result from pre-existing mechanical asymmetries of the biological system, or asymmetries caused by biological signalling during lumenogenesis. In the early, spherical stage of a forming bile canaliculus [35], the junction belt constitutes an example of such a mechanical asymmetry [21], but it remains unknown whether and how this asymmetry is coupled to the sphere-to-tube transition of the bile canaliculus. Moreover, as noted above, during the later extension of tubular bile canaliculi, anisotropic mechanics help to guide the bile canaliculi via anisotropic tension [18] and support them via apical bulkheads [21, 29].

However, such a symmetry breaking could also be spontaneous: Even in the absence of biological signalling or mechanical asymmetries, a non-spherical lumen shape might be favoured energetically over a spherical shape. This spontaneous symmetry breaking might result from active, out-of-equilibrium processes, consistently with instabilities [61, 62] of spherical active surfaces [63]. It could also result, even in thermodynamic equilibrium, from mechanical nonlinearities, as noted previously [53] and as illustrated in a minimal example below. Which of these different mechanisms operate in specific biological systems and their relative contribution to the emergence of non-spherical lumen shapes remains largely unknown, however, not only because the mechanical forces driving lumen formation in specific biological systems are still being investigated actively [4, 10, 15], but even more so because the active and nonlinear mechanical possibilities are poorly understood. The contribution of related mechanical instabilities to more complex shape changes of cysts and lumina such as eversion [64, 65] also remains to be explored more fully.

### Example: symmetry breaking in a mean-field vertex model

**Figure 3.**
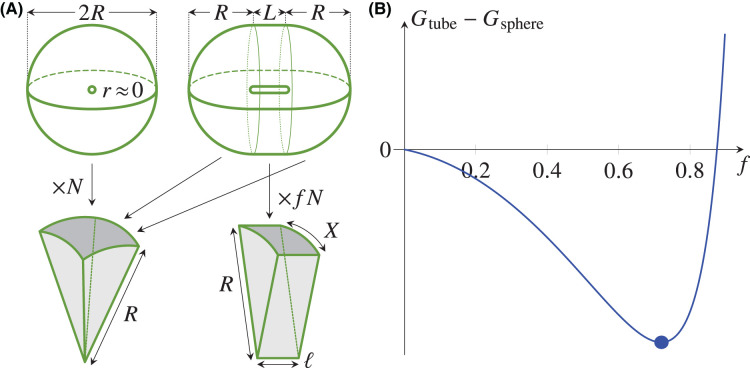
Mechanics of non-spherical lumina: Spontaneous symmetry breaking due to mechanical nonlinearities. (**A**) Spherical cyst of radius R and small lumen radius r≈0, and elongated, tubular cyst consisting of two hemispherical caps of radius R and a cylindrical middle region of radius R and length L. The N cells in the cyst are either square pyramids of side R or cylindrical sectors of radius R, thickness ℓ and length X. A fraction f of the cells are in the cylindrical region. (**B**) Plot of the difference $G_{\text{tube}}-G_{\text{sphere}}$ of the enthalpies of the tubular and spherical cysts, showing a negative minimum value at some f>0, highlighted by the large mark, and illustrating spontaneous symmetry breaking.

We illustrate the possibility of spontaneous symmetry breaking in a minimal model: In the mean-field vertex model description developed above, we consider the limit r→0 of a small lumen or, equivalently, the case of a cyst of thick cells (Figure 3A). In this limit, eqn (6) yields

9 $$G_{\text{sphere}}=4\pi {\left(\frac{3NV}{4\pi }\right)}^{2/3}\left[\Gamma_{b}+\frac{N\Gamma_{\ell }}{2\pi }\cos^{-1}{\left(\sec\frac{\pi }{N}-\tan\frac{\pi }{N}\right)}^{2}\right].\;\left(9\right)$$

We compare this enthalpy to that of a cylindrical tube with hemispherical caps of radius R (Figure 3A). The cylindrical part of the tube has radius R, too, and length L, and contains fN cells, while the caps contain (1−f)N cells, for some fraction f (Figure 3A). The cells in this part are cylindrical sectors of radius R, thickness ℓ, and length X (Figure 3A). They have basal area $A_{b}=X\ell$ and lateral area $A_{\ell }=2[X/(2\pi R)(\pi R^{2})+\ell R]=(X+2\ell )R$. The enthalpy of the cylindrical part is, therefore, $G_{\text{cylinder}}=$ $G_{\text{cylinder}}=\Gamma_{b}(2\pi RL)+\Gamma_{\ell }(\;fN)(X+2\ell )R$, while that of the caps is obtained by replacing N→(1−f)N in eqn (9). Now R,ℓ,L are determined by the volume balance conditions $\frac{4}{3}\pi R^{3}=(1-f)NV$, $\pi R^{2}L=fNV$, and the condition imposing the volume of a single cell, ℓXR/2=V. Hence

10 $$\begin{array}{rl} G_{\text{tube}} & =G_{\text{caps}}+G_{\text{cylinder}}\\ & =4\pi {\left(\frac{3(1-f)NV}{4\pi }\right)}^{2/3}\left[\Gamma_{b}+\frac{(1-f)N\Gamma_{\ell }}{2\pi }\cos^{-1}{\left(\sec\frac{\pi }{(1-f)N}-\tan\frac{\pi }{(1-f)N}\right)}^{2}\right]\\ & \;+2\Gamma_{b}fNV{\left(\frac{3(1-f)NV}{4\pi }\right)}^{-1/3}+fN\Gamma_{\ell }\left[{\left(\frac{3(1-f)NV}{4\pi }\right)}^{1/3}X+\frac{4V}{X}\right], \end{array}$$

which is minimised, in the tension-dominated case $\Gamma_{\ell }>0$, for $X=2[4\pi V^{2}/3(1-f)N{]}^{1/6}$. With this value of X, we find, for f≪1 and N≫1,

11 $$G_{\text{tube}}-G_{\text{sphere}}\approx -\Gamma_{\ell }fV^{2/3}{\left(\frac{N^{7}}{972\pi }\right)}^{1/6}\left(7\sqrt{3}-12\right)<0.\;\left(11\right)$$

This shows that the enthalpy of the tube configuration is smaller than that of the spherical one for any $\Gamma_{\ell }>0$, which is confirmed by a plot of $G_{\text{tube}}-G_{\text{sphere}}$ against f that exhibits a minimum at some non-zero f>0 (Figure 3B). This simple model thus implies spontaneous elongation into a tube due to the mechanical nonlinearities that emerge, at the cyst scale, from single-cell mechanics. This minimal calculation in the limit r→0 cannot of course capture the effect of lumen pressure p or apical tension $\Gamma_{a}$ or apical belt tension Λ on this transition, which likely depends additionally on the assumed cell shapes. This minimal calculation thus serves its double purpose to illustrate the possibility of spontaneous symmetry breaking and to stress that developing more accurate models of this sphere-to-tube transition remains an important challenge for future work.

## Outlook

We have reviewed the formation of epithelial lumina under pressure. We have highlighted the interplay of pressure and mechanical forces in the cells surrounding the lumen that defines the lumen shape.

Focusing on these mechanical forces, we have first illustrated the formation of spherical lumina via the Young–Laplace law in simple models, showing in particular how the lumen-scale tension in the Young–Laplace law can emerge from the mechanics of individual cells. We have stressed that these emergent mechanics will be different, in general, from the classical mechanics of, for example, elasticity. We have also shown how these nonlinear emergent mechanics can lead to a spontaneous symmetry breaking that implies the formation of non-spherical lumina, and we have discussed different additional mechanisms that could be involved in this symmetry breaking.

In this way, our minimal calculations have stressed the need for new theoretical descriptions of the mechanics that emerge at the lumen scale from the anisotropic mechanical properties of individual cells. On the experimental side, testing these descriptions will rely on quantitative measurements of mechanical parameters. While techniques for indirect [16] and more direct [10, 14, 17] pressure measurements and even pressure manipulation [13] are beginning to be established, quantification of the pressure in small lumina, as in the bile canaliculi example system, still poses a challenge. Measuring the anisotropic tensions of different cell surfaces poses an even greater challenge. For example, the lateral tension that is represented by the single parameter $\Gamma_{\ell }$ in the vertex model (5) includes both positive contributions from the lateral cell cortex and negative ones from cell–cell adhesion [53]. This stresses the particular need to approaches that allow quantification of multiple parameters in the same experimental context.

Meanwhile, in the confines of this short review, we could not discuss in detail the physics of osmotic and hydraulic flows that pump fluid into the lumen and hence build up the pressure that is opposed by the mechanical forces of the surrounding cells, but, as already noted above, their physics have been reviewed authoritatively and excellently elsewhere [39]. We close by noting that theories of flexoelectricity that describe the coupling between these solute flows, electricity, and the mechanics of the surrounding cells discussed here are beginning to emerge [66, 67].

## Perspectives

- The formation of pressurised epithelial lumina in development and disease relies on a balance of the pressure in the lumen and the mechanics of the cells surrounding it.
- Much of the current work in biophysics and mechanobiology has focused on descriptions of spherical lumina, while the physical mechanisms underlying the formation of non-spherical lumina has received less attention.
- To understand the emergence of non-spherical lumen shapes, future studies will need to disentangle the contribution of biological signalling and anisotropic biological structures on the one hand, and symmetry breaking of spherical lumina via nonlinear and active mechanical processes on the other.

## Abbreviations

2D: two-dimensional
3D: three-dimensional
MDCK: Madin–Darby canine kidney
